# Outcomes of Metabolic and Bariatric Surgery in Patients with Inflammatory Bowel Disease: A Long-Term Retrospective Analysis

**DOI:** 10.3390/jcm14020402

**Published:** 2025-01-10

**Authors:** Adi Litmanovich, Jonathan Benjamin Yuval, Elena Donata Agostini, Lior Orbach, Yehuda Kariv, Meir Zemel, Guy Lahat, Adam Abu-Abeid

**Affiliations:** 1 Division of General Surgery, Tel Aviv Sourasky Medical Center, 6 Weizman St., Tel Aviv 6423906, Israel; adidaae@gmail.com (A.L.); jyuval@gmail.com (J.B.Y.); elena.a_92@live.it (E.D.A.); kariv_y@yahoo.com (Y.K.); meirz@tlvmc.gov.il (M.Z.); guyla@tlvmc.gov.il (G.L.); 2 The Faculty of Medical and Health Sciences, Tel Aviv University, Tel Aviv 6997801, Israel; lioro@tlvmc.gov.il; 3 Colorectal Surgery Unit, Division of General Surgery, Tel Aviv Sourasky Medical Center, 6 Weizman St., Tel Aviv 6423906, Israel; 4 Bariatric Surgery Unit, Division of General Surgery, Tel Aviv Sourasky Medical Center, 6 Weizman St., Tel Aviv 6423906, Israel

**Keywords:** metabolic surgery, bariatric surgery, weight loss surgery, inflammatory bowel disease, Crohn’s disease, ulcerative colitis

## Abstract

**Background**: Metabolic and bariatric surgery (MBS) is a well-established treatment for severe obesity, yet its effects in patients with inflammatory bowel disease (IBD) are not well understood. MBS in this population presents unique challenges, including the potential for exacerbating inflammatory disease activity and causing complications such as malnutrition and medication malabsorption. This study aims to assess the long-term outcomes of MBS in IBD patients, focusing on both metabolic outcomes and its impact on the course of IBD. **Methods**: A retrospective analysis was conducted on 20 patients with IBD who underwent MBS at a tertiary center between 2005 and 2019. Data on baseline characteristics, surgical procedures, complications, weight loss, resolution of obesity-related diseases, and IBD-related outcomes were collected. **Results**: The cohort, primarily female (65%), had a mean preoperative body mass index (BMI) of 40.8 kg/m^2^. The MBS procedures performed were sleeve gastrectomy (n = 9), Roux-en-Y gastric bypass (n = 6), one-anastomosis gastric bypass (n = 2), and Laparoscopic Adjustable Gastric Banding (n = 3). No major 30-day complications were recorded. At a median follow-up of 91 months, the mean BMI decreased by 9.5 kg/m^2^, with satisfactory outcomes in terms of resolution of obesity-related diseases. IBD activity scores increased postoperatively, particularly in Crohn’s disease (CD) patients, although these changes were not statistically significant. In addition, 30% of patients were hospitalized due to IBD exacerbation, and 15% required surgical intervention for IBD. **Conclusions**: MBS is an effective treatment for severe obesity and its related diseases in IBD patients. While encountering no major complications or mortality, some long-term complications were observed, with a possible increase in IBD activity, particularly in CD patients. Ongoing challenges, such as the risk of malnutrition, medication malabsorption, and postoperative IBD exacerbations, necessitate careful long-term follow-up.

## 1. Introduction

Metabolic and bariatric surgery (MBS) is considered the most effective treatment for patients with severe obesity [1]. Its popularity has increased rapidly since the beginning of the 21st century, and the indications for MBS were recently updated to include specific patient considerations [2,3]. The updated guidelines for MBS are for patients with body mass index (BMI) ≥ 35 kg/m^2^, regardless of the presence, absence, or severity of obesity-related conditions. MBS should be considered in individuals with a BMI of 30–34.9 kg/m^2^ who do not achieve substantial or durable weight loss or improvement in obesity-related conditions with conservative methods. Along with the advantages of MBS, which include durable long-term weight loss, improvement and/or resolution of severe obesity-related conditions, and reduced mortality, there are chronic conditions that may be encountered postoperatively during patient follow-up, including insufficient weight loss/weight regain, reflux, malnutrition, and more. These may need further investigation and may even require revisional surgery. The development of these chronic complications could be related to many factors, including surgical technique, type of MBS (restrictive vs. hypo absorptive), and patient-related factors including age, gender, BMI, compliance with medical recommendations, and physical status. One area of practice, that remains uncertain, is MBS considerations in patients with inflammatory bowel disease (IBD).

IBD is a chronic condition with a multifactorial etiology, affecting approximately 0.5% of the Western population [4]. The two main subtypes of IBD are Crohn’s disease (CD) and ulcerative colitis (UC). These diseases can range from mild to severely debilitating and may also cause malnutrition and cachexia. Patients may require multiple lines of medical and surgical treatment. Severe obesity rates among patients with IBD are increasing and range from 15% to 40% [5]. MBS in patients with IBD has been previously reported, and the type of procedure offered varied according to several factors, including BMI, disease severity and location, patient compliance, and more. However, there are many uncertainties and challenges in managing IBD patients before MBS, including determining the optimal surgical procedure, assessing the impact of MBS on IBD symptoms, and addressing the risks of both early and long-term complications, such as malnutrition and medication malabsorption, particularly with hypo-absorptive procedures.

The purpose of this study is to evaluate the short- and long-term outcomes of patients with IBD undergoing MBS. We hypothesize that these patients achieve satisfactory metabolic and nutritional outcomes, with no negative impact on the course of their IBD.

## 2. Methods

This is a retrospective analysis of a prospectively maintained patient database. All patients underwent MBS in a single tertiary university hospital between 2005 and 2019. Patients diagnosed with IBD prior to MBS were captured and made up the study cohort.

### 2.1. Patients

Data retrieved included patients’ baseline characteristics, including age, gender, BMI, previous MBS, and obesity-related medical conditions—diabetes mellitus (DM), hypertension (HTN), hyperlipidemia (HL), Obstructive sleep apnea (OSA), and gastroesophageal reflux disease (GERD). IBD-related data included age at diagnosis, type of IBD (CD, UC, or indeterminate colitis IC), medical treatments prior to MBS, and prior surgical interventions for IBD. When available, Crohn’s disease activity index (CDAI) and Mayo score were recorded to denote the severity of symptoms in CD and UC, respectively. Perioperative data collected included the type of MBS performed, intraoperative complications, 30-day complications, length of stay, readmissions, and reoperations.

### 2.2. Surgical Technique

All procedures were performed laparoscopically using a standardized approach by one of the surgeons in the MBS unit. Venous thromboembolism prophylaxis (subcutaneous heparin, 5000 units) and antibiotic prophylaxis (intravenous cefazolin, 2–3 g) were routinely administered. The abdomen was insufflated using a Veress needle with carbon dioxide to a pressure of 15 mmHg, and at least five laparoscopic trocars were inserted. Technical steps for each procedure were adapted from a previous study [6] and are outlined below:

### 2.3. One-Anastomosis Gastric Bypass (OAGB)

Dissection was performed below the level of the crow’s foot at the lesser curvature of the stomach, extending to the lesser sac. A long and narrow gastric pouch was created using multiple linear stapler firings (Echelon -Ethicon Endo-Surgery Inc., Cincinnati, OH, USA or End-GIA Covidien/Medtronic Inc., Minneapolis, MN, USA) guided by a 34–36 Fr bougie. The ligament of Treitz was identified, and the bowel was measured 180–200 cm distally. A linear stapled anastomosis was then performed between the gastric pouch and the jejunal loop, with the opening manually sutured using a barbed suture. A routine blue dye leak test was conducted.

### 2.4. Sleeve Gastrectomy (SG)

The greater curvature of the stomach was mobilized approximately 4 cm proximal to the pyloric sphincter, extending to the angle of His and exposing the left crus of the diaphragm. A 36–40 Fr bougie was used for calibration along the lesser curvature. The stomach was then transected using multiple linear staplers, and the specimen was removed through one of the working ports. Staple line reinforcement was not routinely used. A blue dye leak and patency test was performed.

### 2.5. Roux-en-Y Gastric Bypass (RYGB)

A short gastric pouch was created using a linear stapler after dissection along the proximal lesser curvature, reaching the lesser sac. The ligament of Treitz was identified, and the bowel was transected 50–100 cm distally to create the bilio-pancreatic limb. Stapled gastrojejunal and jejunojejunal anastomoses were constructed, creating a 120–150 cm alimentary limb. In a case of conversion from Laparoscopic Adjustable Gastric Banding (LAGB), the band was removed during the procedure.

### 2.6. Laparoscopic Adjustable Gastric Banding (LAGB)

The gastrohepatic ligament was dissected at the pars flaccida of the lesser omentum, and a tunnel was created posterior to the stomach wall toward the angle of His. The band was introduced and locked anteriorly, then fixed with nonabsorbable sutures. The tube was tunneled to the left lateral abdominal wall, and the port was anchored to the fascia.

### 2.7. Follow-Up

Follow-up data included BMI and total weight loss (TWL) at the last visit. Resolution of obesity-related conditions (DM, HTN, and HL) was recorded (defined by cessation of medication). Data on chronic complications and reoperations related to MBS were also collected. IBD severity was recorded using the CDAI for CD and the Mayo score for UC, when available [7,8,9]. Data on hospitalizations, medications at last follow-up, and operations for IBD exacerbations were captured.

### 2.8. Statistical Analysis

Statistical analysis was performed using SPSS version 29 (IBM SPSS Statistics, Chicago, IL, USA). Continuous data are presented as mean ± standard deviation or median (range), as appropriate. Categorical data are presented as numbers (percentages). Differences between groups were assessed using Student’s *t*-test, with *p*-values < 0.05 considered statistically significant.

### 2.9. Ethical Approval

The study was approved by our medical center’s Institutional Review Board (IRB) and conducted in compliance with the ethical standards of the institutional and/or national research committee and with the 1964 Declaration of Helsinki and its later amendments or comparable ethical standards. Due to the retrospective nature of the study, informed consent was waived by the IRB.

## 3. Results

During the study period, 20 patients with IBD underwent MBS at our center. These included 10 patients with CD, 9 with UC, and 1 with IC. Baseline characteristics of the study cohort are presented in Table 1. The cohort predominantly consisted of women (65%) with a mean age of 40.3 ± 11.3 years and a mean BMI of 40.8 ± 4.0 kg/m^2^. One patient (5%) had a history of previous MBS. Prior to MBS, nine patients (45%) were on chronic medical treatment for IBD. At the time of MBS, the mean CDAI score for CD patients was 62.3 ± 35.7 (range 22–134), and the mean Mayo score for UC patients was 2.7 ± 2.4 (range 0–6).

Operative and perioperative data are shown in Table 2. SG was performed in nine patients (45%), RYGB in six patients (30%), OAGB in two patients (10%), and laparoscopic LAGB in three patients (15%). There were no intraoperative complications or major 30-day complications. One patient (5%) required readmission due to dysphagia and dehydration with a gradual and spontaneous resolution.

**Figure 1:**
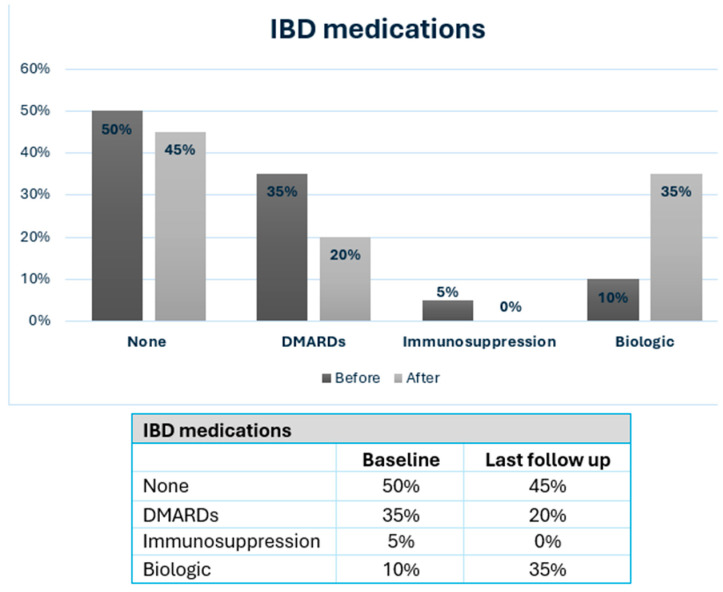
IBD patients consuming medication at baseline and at last follow-up after MBS. IBD: inflammatory bowel disease; DMARDs: Disease-Modifying Anti-Rheumatic Drugs; MBS: metabolic and bariatric surgery.

Long-term outcomes of MBS and IBD are detailed in Table 3, with a median follow-up of 91 months (mean 96.3 ± 41.1 months). The mean BMI at the last follow-up was 31.2 ± 7.3 kg/m^2^, reflecting a mean reduction of 9.5 ± 7.9 kg/m^2^. IBD-related outcomes were available in 14 patients (70% of cohort). At the last follow-up, 11 patients (55%) were on chronic medical treatment (Figure 1), 6 patients (30%) were hospitalized due to IBD exacerbations, and 3 patients (15%) underwent bowel resections related to IBD. The mean CDAI at follow-up was 135.2 ± 76.2 (range 46–269) vs. 62.3 ± 35.7 at baseline, (*p* = 0.055). The mean Mayo score for UC at last follow-up was 3.0 ± 1.6 vs. 2.7 ± 2.4 at baseline, (*p* = 0.411).

**Figure 2:**
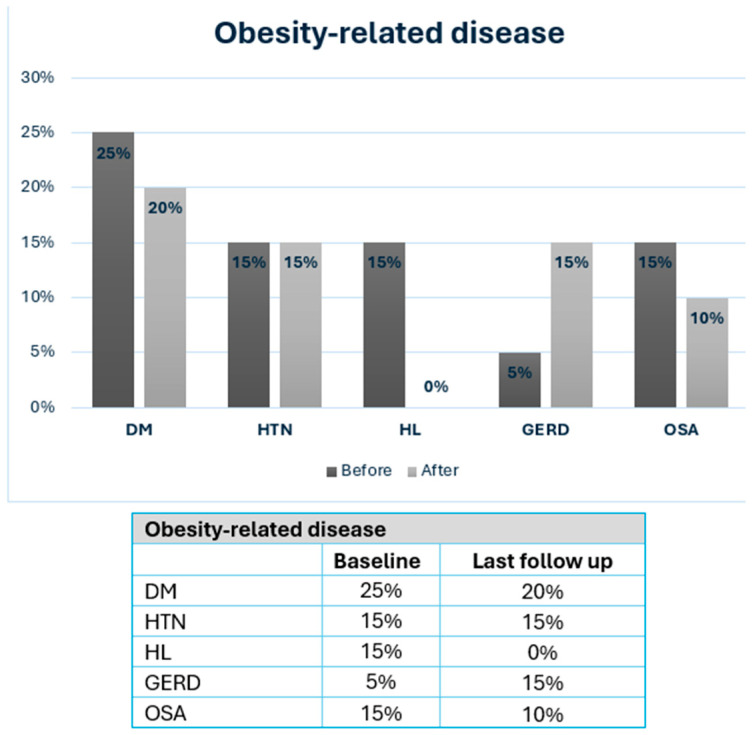
Severe obesity-related disease in IBD patients at baseline and after MBS. MBS: metabolic and bariatric surgery; DM: diabetes mellitus; HTN: hypertension; HL: hyperlipidemia; GERD: gastroesophageal reflux disease; OSA: Obstructive sleep apnea.

Obesity-related diseases at baseline and after MBS are shown in Figure 2. At last follow-up, four patients (20%) had DM, three patients (15%) had HTN, three patients (15%) had GERD, two patients (10%) had OSA, and none had hyperlipidemia. Chronic micronutrient deficiencies were present in 9 patients (45%) and included vitamin D deficiency (n = 4), iron deficiency (n = 6), B12 deficiency (n = 5), and 1 patient had severe protein energy malnutrition which required revision to normal anatomy after RYGB.

## 4. Discussion

In this case series with a review of the literature, we presented our experience with MBS in IBD patients. We observed that IBD activity scores increased after surgery, especially for CD patients, though this did not reach statistical significance. The mean CDAI for CD increased from 62.3 ± 35.7 to 135.2 ± 76.2 (*p* = 0.055), while the mean Mayo score for UC increased from 2.7 ± 2.4 to 3.0 ± 1.6 (*p* = 0.411). Although not reaching statistical significance, these changes coincided with increased therapeutic needs: the percentage of patients needing IBD medication increased from 50% to 55%, with 35% requiring biologic therapy (Figure 1). In addition, a significant minority of IBD patients required hospitalization (30%) or surgery (15%) for IBD exacerbation following MBS. The literature on MBS’s impact on IBD presents varying outcomes. Table 4 summarizes the published studies of MBS in IBD patients, highlighting both metabolic outcomes and the IBD disease course. Some studies reported exclusively favorable [10,11,12,13,14] or predominantly favorable results [15,16,17], while others have described more negative outcomes [18,19]. Most studies, however, described mixed effects of MBS on the clinical course of IBD [20,21,22,23,24,25,26,27,28]. All studies consistently demonstrated favorable metabolic outcomes, measured by significant postoperative BMI reduction, TWL, or excess weight loss (EWL). A detailed description of all studies is shown in Supplementary Table S1.

Similar to these results, at a median follow-up of 91 months, we demonstrated meaningful metabolic improvements, with a mean BMI reduction of 9.5 + 7.9 kg/m^2^ and a mean last follow-up BMI of 31.2 ± 7.3 kg/m^2^, alongside a resolution or improvement in most obesity-related diseases (Figure 2). The prevalence of DM decreased from 25% to 20%, OSA decreased from 15% to 10%, and HL was completely resolved—findings consistent with previous reports in IBD patients [29].

Overall IBD symptoms worsened in the cohort throughout the study. We found a non-significant trend showing worsening symptoms in both UC and CD, measured by Mayo score and CDAI, respectively. We believe these findings are meaningful even though they were not statistically significant and could help in tailoring MBS to individual patients with IBD. The interplay between IBD and MBS is complex: while severe obesity exacerbates the inflammatory state in IBD through elevated pro-inflammatory adipokines [30] and altered gut microbiota, weight reduction can help mitigate these effects [10,20,21,22,23]. These mechanisms, combined with postoperative changes—including hormonal fluctuations, vitamin deficiencies, bacterial overgrowth, and altered intestinal epithelium—may also exacerbate existing IBD [31] or trigger de novo IBD [32,33]. Kiasat et al. [34] report an 80% increased risk of developing CD and 170% increased risk of developing unclassified IBD after RYGB, and an 80% increased risk of developing UC after SG. Similarly, Allin et al. [35] found an increased risk of de novo CD after MBS, particularly in women, but no association between MBS and UC. Conversely, Kochar et al. [36] reported a lower risk of de novo IBD after MBS, suggesting that effective severe obesity management through surgical or medical interventions might be associated with a reduced risk of developing de novo IBD compared to a control group.

Our findings underscore several challenges specific to the IBD population undergoing MBS. Postoperatively, 30% of patients were hospitalized due to IBD exacerbations, and 15% required surgical intervention due to IBD exacerbation. Chronic malnutrition and hypo-absorption of medications remain concerns as nine patients (45%) in our cohort had some form of malnutrition during follow-up, with one patient requiring MBS surgical revision. These findings emphasize the importance of stringent nutritional and clinical follow-up to address deficiencies in vitamins (e.g., B12, iron, and fat-soluble vitamins) and ensure the efficacy of IBD medications. Consistent with prior research, SG may offer a safer profile for IBD patients by minimizing risks of malabsorption while delivering effective weight loss outcomes [10,37], especially in CD patients [25].

When tailoring the most suitable MBS for IBD patients, some would recommend performing strictly restrictive procedures since these are possibly associated with fewer nutritional complications than hypo-absorptive procedures. In our study, most patients underwent SG (45%), followed by RYGB (30%), OAGB (10%), and LAGB (15%), with a good perioperative safety profile and no major complications. The cohort is too small to compare the MBS and IBD-related outcomes between different surgical procedures. In our opinion, when selecting the type of MBS, many factors should be taken into consideration, including age, BMI, IBD characteristics (type, location, behavior, and severity), route of absorption of IBD medications, prior IBD surgeries, remaining total small bowel length, and more. MBS should be individualized according to these factors. Larger cohort studies are needed to better define the correct approach for these patient populations.

This study has many limitations, including its retrospective design, small sample size representing a case series, variation in the types of MBS performed, and lack of a control group. We did not perform a comparative analysis between different types of MBS due to the low number of patients in each subgroup. In addition, 30% of patients were not available for long-term follow-up, which may skew the data. Despite these limitations, some strengths are worth mentioning, including a relatively long-term median follow-up of 91 months and the use of detailed indices for IBD severity, such as the CDAI and Mayo scores. These factors enhance the reliability and relevance of the findings, emphasizing the need for future prospective studies to clarify the long-term effects of MBS in IBD patients.

## 5. Conclusions

This study demonstrates that MBS is a viable and effective treatment for patients with IBD and severe obesity, providing favorable metabolic outcomes with a potential, though not statistically significant, exacerbation of CD.

Despite these positive outcomes, the study highlights critical considerations for IBD patients, including the risk of postoperative complications such as IBD exacerbations, hospitalizations, and bowel resections, as well as the challenges associated with chronic malnutrition and hypo-absorption of medications, particularly in hypo-absorptive procedures. Future prospective studies with larger cohorts are needed to validate these results, optimize surgical approaches, and refine postoperative care strategies for this unique patient population.

## Figures and Tables

**Table 1:** Baseline characteristics.

	Mean	Standard Deviation	n	(%)
**Patients:**			20	
Female			13	65%
Age	40.4	11.3		
BMI	40.8	4.0		
**Obesity-related disease:**				
DM			5	25%
HTN			3	15%
HL			3	15%
GERD			1	5%
OSA			3	15%
Previous MBS			1	5%
**IBD status**				
CD			10	50%
UC			9	45%
Indeterminate			1	5%
Age at IBD diagnosis	36.4	10.7		
CD CDAI score	62.3	35.7		
UC Mayo score	2.7	2.4		
**IBD medical treatment**				
None			10	50%
DMARDs *			7	35%
Immunosuppression			1	5%
Biologic			2	10%

BMI: body mass index; DM: diabetes mellitus; HTN: hypertension; HL: hyperlipidemia; GERD: gastroesophageal reflux disease; OSA: Obstructive sleep apnea; MBS: metabolic bariatric surgery; IBD: inflammatory bowel disease; CD: Crohn’s disease; UC: ulcerative colitis; CDAI: Crohn’s Disease Activity Index; DMARDs: Disease-Modifying Anti-Rheumatic Drug (* included mesalamine (n = 6) and sulfasalazine (n = 1)).

**Table 2:** Operative and perioperative data.

	n	(%)
Surgery performed		
SG	9	45%
RYGB	6	30%
OAGB	2	10%
LAGB	3	15%
Intraoperative complications	0	0%
30-day readmission	1	5%
Major complications	0	0%
Perioperative mortality	0	0%

SG: sleeve gastrectomy; RYGB: Roux-en-Y gastric bypass; OAGB: one-anastomosis gastric bypass; LAGB: Laparoscopic Adjustable Gastric Banding.

**Table 3:** Long-term outcomes.

	Mean	Standard Deviation	n	(%)
MBS-related outcomes				
Time of F/U (months)	96.3	41.1		
BMI (kg/m^2^)	31.2	7.3		
BMI point reduction	9.5	7.9		
IBD-related outcomes *				
IBD-related Hospitalizations			6	30%
Surgical treatment due to IBD exacerbation			3 *	15%
CD CDAI score	135.2	76.2		
UC Mayo score	3	1.63		
Chronic micronutrient deficiencies			9	45%
Severe protein energy malnutrition			1	5%

MBS: metabolic and bariatric surgery; BMI: body mass index; IBD: inflammatory bowel disease; CD: Crohn’s disease; UC: ulcerative colitis; CDAI: Crohn’s Disease Severity Index. * 2 patients with CD and 1 indeterminate colitis.

**Table 4:** Review of studies describing outcomes of MBS on IBD.

Reference	Number of Patients Total (CD/UC/IC)	MBS Performed				F/U Time (Months)	Weight Loss			IBD Outcomes
		SG	RYGB	AGB	Other		BMI change (mean ± SD)	Mean TBWL %	Mean EWL %	
Desai et al., 2024 [10]	482 (289/193/0)	328 (68%)	154 (32%)	0	0	24 (IBD), 12 (Weight loss)	42.1 ± 7.07 to 36 ± 6.8 (SG) and 31 ± 6.4 (RYGB)	N/A	N/A	Favorable or mostly favorable outcomes
Ferrer-Márquez et al., 2023 [20]	41 (22/18/1)	31 (75%) CD 81.8% UC 66.6%	1 (2.4%)	0	9 OAGB (22%)	12	45.8 ± 6.1 to 29.5 ± 4.7	33.9 ± 9.1	N/A	Mixed outcomes
Seidemann et al., 2023 [11]	1 (1/0/0)	1	0	0	0	36	N/A	23	49	Favorable or mostly favorable outcomes
Corbiere et al., 2023 [18]	587 (326/261/0)	476 (81.1%)	86 (14.6%)	20 (3.4%)	5 (0.9%)	24	N/A	N/A	N/A	Mostly negative outcomes
Reenaers et al., 2022 [24]	85 (64/21/0)	72 (81.8%)	3 (3.4%)	12 (13.6%)	0	34	N/A	UC—23.3 ± 1 CD—23.4 ± 12	N/A	Mixed outcomes
McKenna et al., 2020 [15]	31 (10/20/1)	14 (46.7%) 7 UC 7 CD	14 (46.7%) 9 UC 4 CD 1 IC	4 (UC)	0	32.4	BMI change 12.5 (1 year) 10.3 (2 years)	29.8 (1 year), 23.6 (2 years)	62.9 (1 year), 57.4 (2 years)	Favorable or mostly favorable outcomes
Braga Neto et al., 2020 [21]	47 (18/29/0) Matched case controls (12 CD,13 UC)	8 (17%) 5 UC 3 CD	28 (59.6%) 16 UC 12 CD	8 (17%) 6 UC 2 CD	3 (6.4%) 2 UC 1 CD	92.3	CD—from 49.5 to 34. UC—from 43 to 32.8	N/A	N/A	Mixed outcomes
Heshmati et al., 2019 [25]	54 (31/23/0)	35 (64.8%) 12 UC 23 CD	19 (35.2%) 11 UC 8 CD	0	0	SG—26 RYGB—96	1 year F/U: SG—from 44.9 ± 7.3 to 33.4 RYGB—from 48.5 ± 7.7 to 43.5	1 year F/U: SG—23 RYGB—31	N/A	Mixed outcomes
Hudson et al., 2019 [12]	13 (9/4/0)	9 (69.2%) 1 UC 8 CD	3 (23.1%) 2 UC 1 CD	1 (7.7%) UC	0	13 ± 7	1 year F/U: from 48.1 ± 2.9 to 31.7 ± 1.5	N/A	N/A	Favorable or mostly favorable outcomes
Raziel et al., 2019 [26]	8 (8/0/0)	8 (100%)	0	0	0	N/A	From 44.3 ± 2.4 to 28.7 ± 1.3	N/A	N/A	Mixed outcomes
Sharma et al., 2018 [27]	493 (248/245/0)	233 (47%)	176 (35.5%)	87 (17.5%)	0	N/A	N/A	N/A	N/A	Mixed outcomes
Aelfers et al., 2018 [17]	45 (27/15/3)	23 (51%) 5 UC 16 CD 2 IC	9 (20%) 4 UC 4 CD 1 IC	6 (13%) 1 UC 5 CD	7 (16%) 4 UC 3 CD	46.8 ± 36	N/A	at 2 years F/U: 26.6 ± 12.2	at 2 years F/U: 62.9 ± 31	Mixed outcomes
Honoré et al., 2018 [13]	8 (8/0/0)	8 (100%)	0	0	0	12	N/A	N/A	at 1 year F/U: 56.5%	Favorable or mostly favorable outcomes
Aminian et al., 2016 [22]	20 (7/13/0)	9 (45%) 5 UC 4 CD	8 (40%) 7 UC 1 CD—conversion from AGB	3 (15%) 1 UC 2 CD	0	34.6 ± 21.7	1 year F/U: 14.3 ± 5.7	N/A	at 1 year F/U: 58.9 ± 21.1	Mixed outcomes
Colombo et al., 2015 [16]	6 (5/1/0)	5 (83.3%) 1 UC 5 CD	0	0	1 (16.7%) VBG	57.8 ± 29.8	from 40.6 ± 1.7 to 26.8 ± 1.1	N/A	74.5 ± 11.2	Favorable or mostly favorable outcomes
Keidar et al., 2014 [28]	10 (8/2/0)	9 (90%)	0	1 (10%)	0	46 (9–67)	from 42.6 to 29	N/A	71	Mixed outcomes
Ungar et al., 2013 [14]	4 (4/0/0)	4 (100%)	0	0	0	22.8 ± 18	at 6–12 months: 45 ± 5.3 to 32.9 ± 4.3	N/A	60.3 ± 13.7	Favorable or mostly favorable outcomes
Moum et al., 2010 [19]	1 (1/0/0)	0	1 (100%)	0	0	8	from 45 to 32.4	N/A	N/A	Mostly negative outcomes
Lascano et al., 2006 [23]	1 (0/1/0)	0	1 (100%)	0	0	24	from 57 to 31	N/A	80	Mixed outcomes

CD: Crohn’s disease; UC: ulcerative colitis; IC: indeterminate colitis; MBS: metabolic and bariatric surgery; IBD: inflammatory bowel disease; SG: sleeve gastrectomy; RYGB: Roux-en-Y Gastric Bypass; AGB: Adjustable Gastric Banding; BMI: body mass index; TBWL: Total Body Weight Loss; EWL: Estimated Weight Loss.

## Data Availability

The raw data supporting the conclusions of this article will be made available by the authors on request.

## Supplementary Materials

The following supporting information can be downloaded at: https://www.mdpi.com/article/10.3390/jcm14020402/s1, Table S1. Review of studies evaluating MBS outcomes on IBD patients: detailed postoperative outcomes of bariatric surgery on IBD course.
