# Supplementary material for: Outcomes of Metabolic and Bariatric Surgery in Patients with Inflammatory Bowel Disease: A Long-Term Retrospective Analysis
Source: J Clin Med. 2025 Jan 10;14(2):402. doi: 10.3390/jcm14020402 (PMC11765963; doi:10.3390/jcm14020402)
Supplement: Supplementary file 1 [file jcm-14-00402-s001.zip › jcm-3369426-supplementary.pdf]

Supplementary Table S1. Review of studies evaluating MBS outcomes on IBD patients: detailed postoperative outcomes of bariatric surgery on IBD course

Reference	IBD outcomes
**Desai et al.** ***2024*** **[10]**	Reduced IBD complications, need for IV/oral steroids, biological or small molecule therapy initiation
**Ferrer-Márquez et al.** ***2023*** **[20]**	- 34.2% of patients were able to reduce their IBD treatment<br>- 13.2% required increased treatment, 1 CD patient required additional surgery (perianal abscess drainage) due to flare-up<br>- One patient (2.6%) required surgery (perianal abscess drainage) secondary to a CD flare-up
**Seidemann et al.** ***2023*** **[11]**	Complete resolution of CD symptoms and no relapses
**Corbiere et al.** ***2023*** **[18]**	- Eighty-six (14.65%) IBD patients had at least one unplanned readmission for IBD management<br>- CD patients had a higher risk of IBD-related readmissions compared to UC
**Reenaers et al.** ***2022*** **[24]**	- Sixty-four patients (75%) did not require any changes to their IBD medications<br>- Three patients (3.5%) had treatment de-escalation<br>- Fifteen patients (17.6%) required treatment escalation for loss of response (including one salvage colectomy due to UC flare)<br>- Two patients (2.3%) developed new anoperianal lesions<br>- Five patients (5.9%) were hospitalized for IBD flare w/o surgery
**McKenna et al.** ***2020*** **[15]**	- No IBD flares requiring surgery<br>- Seven patients with no change in Immunosuppressive medication<br>- Two patients decreased medication—from biologic treatment to none<br>- One patient increased medication—from none to immunomodulator
**Braga Neto et al.** ***2020*** **[21]**	- Two patients (one UC, one CD) were able to discontinue biologic medications<br>- Two patients (one UC, one CD) had disease progression<br>Compared to matched controls (25 IBD w/o MBS):<br>- Fewer IBD-related complications, less need for rescue corticosteroids, fewer IBD-related surgeries
**Heshmati et al.** ***2019*** **[25]**	- 100% of UC patients undergoing SG had improvement or no change in IBD-medical treatment<br>- 9% of UC patients undergoing RYGB had treatment escalation<br>- 4% of CD patients undergoing SG and 37.5% undergoing RYGB had treatment escalation<br>- Five patients developed IBD-related complications, two required IBD surgery and one RYGB reversal
**Hudson et al.** ***2019*** **[12]**	- At 12 months none of the patients had an IBD flare<br>- Two patients discontinued IBD medical therapy
**Raziel et al.** ***2019*** **[26]**	- One patient had decreased CD symptoms<br>- One patient with mild disease had a very severe relapse 10 months post-surgery

Reference	IBD outcomes
**Sharma et al; *2018* [27]**	Compared to controls (14,826 IBD w/o MBS):<br>- Renal failure, undernutrition, and fistulae were significantly lower<br>- Strictures were significantly higher in UC but not CD patients
**Aelfers et al. *2018* [17]**	Three IBD patients (one UC, two CD) experienced an exacerbation of their IBD, more than 1 year after surgery
**Honoré et al. *2018* [13]**	All patients remained in remission, with no changes in CD medication, no hospital admissions due to CD, and no operative intervention for CD
**Aminian et al. *2016* [22]**	- Nine out of ten patients had improvement in their IBD status (characterized by discontinuation or significant reduction in maintenance IBD medications, subjective improvement of IBD-related symptoms)<br>- Two patients experienced acute flare-up of UC early after MBS<br>- One patient initially improved for 3 years following AGB but later developed acute flares, coinciding with significant weight regain
**Colombo et al. *2015* [16]**	- All patients were able to discontinue steroids, and 2 patients were able to halve their azathioprine dosage<br>- Significant reduction in BMI, CRP levels, WBC, and SBP<br>- Four out of five CD patients were in endoscopic remission at 1 year<br>- One CD patient had complete healing of rectum and perianal fistulas, remained asymptomatic and in remission<br>- One CD patient experienced a clinical recurrence which was treated with adalimumab
**Keidar et al. *2014* [28]**	- Seven patients had no IBD exacerbations<br>- Three patients were able to stop their 5-ASA treatment<br>- Three patients experienced mild exacerbation of Crohn's disease<br>- One patient had to start 5-ASA due to disease exacerbation
**Ungar et al. *2013* [14]**	All patients remained in remission, no worsening of CD, no increase in CD-related complications, no increase in CD medication
**Moum et al. *2010* [19]**	Shortly after the BS, a disease flare-up occurred, including worsening symptoms, elevated calprotectin, and inflammatory markers
**Lascano et al. *2006* [23]**	- Patient reported improvement of IBD symptoms, resolution of associated pyoderma gangrenosum, and decrease in medication requirements<br>- Active disease in postoperative colonoscopy and biopsy

IBD inflammatory bowel disease; *CD* Crohn's disease; *UC* ulcerative colitis; *MBS* metabolic and bariatric surgery; *SG* sleeve gastrectomy; *RYGB* Roux-en-Y gastric bypass; *AGB* adjustable gastric banding
